# Case Report: Transcatheter Closure of Ruptured Sinus of Valsalva With Ventricular Septal Defect Occluder in a 3-Year-Old Child After Repair of Ventricular Septal Defect

**DOI:** 10.3389/fped.2021.751163

**Published:** 2021-09-30

**Authors:** Xueyang Gong, Jundao Wang, Luyao Wei, Tianli Zhao, Shijun Hu

**Affiliations:** Department of Cardiovascular Surgery, The Second Xiangya Hospital, Central South University, Changsha, China

**Keywords:** sinus of Valsalva, congenital heart disease, retrograde approach, transcatheter closure, echocardiogram

## Abstract

Sinus of Valsalva aneurysm (SVA) is a rare cardiac anomaly that can undergo spontaneous rupture into other cardiac chambers or the pericardial space. A ruptured SVA has a very poor prognosis with high morbidity and mortality. These aneurysms often present as a rupture from the right coronary sinus into the right ventricle. Transcatheter closure has become an effective alternative to surgical treatment. However, it has been rarely reported in patients after ventricular defect repair in the past. We here describe a 3-year-and-3-month-old boy who was found to have a ruptured sinus of Valsalva. He underwent surgical closure of a ventricular septal defect at the age of 2 months, which occurred in the non-coronary sinus (NCS) and ruptured into the right atrium. We successfully occluded the ruptured sinus of Valsalva with a ventricular septal occluder.

## Introduction

Sinus of Valsalva (SOA) rupture is a rare cardiovascular disease, and it occurs in the presence of sinus of Valsalva aneurysm (SVA) (1). SVA is a rare cardiac anomaly which could be congenital or acquired (2–4). The cases of aortic sinus rupture in the absence of aneurysm were even rarer, which may lead to symptoms such as aortic regurgitation and heart failure, which were the same as the symptoms of SAV. The aneurysms most commonly arise from the right coronary sinus of Valsalva and most frequently rupture into the right ventricle. However, we here report a case of the sinus of Valsalva that ruptured after ventricular septal defect repair, which occurred in the NCS and ruptured into the right atrium. We successfully occluded the ruptured SOA with a ventricular septal occluder.

## Case Description

**Figure 1:**
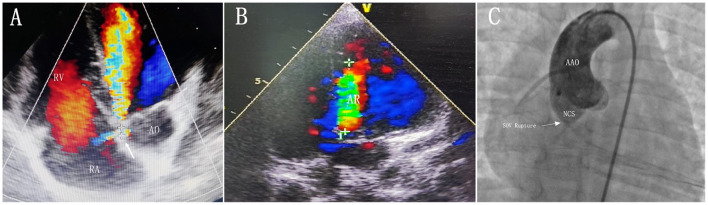
**(A)** Transthoracic echocardiogram obtained in the five-chamber view showing that the SOV rupture occurr in the non-coronary sinus and rupture into the right atrium. **(B)** Transthoracic echocardiogram obtained in the apical long axis view showing a mild-to-moderate aortic regurgitation. **(C)** Aortography showing significant shunting between NCS and the right atrium. The defect diameter of 3 mm was measured by aortography. AR, aortic regurgitation; AO, aorta; AAO, ascending aorta; RA, right atrium; RV, right ventricle; NCS, non-coronary sinus of Valsalva; SOV rupture, Sinus of Valsalva rupture.

**Figure 2:**
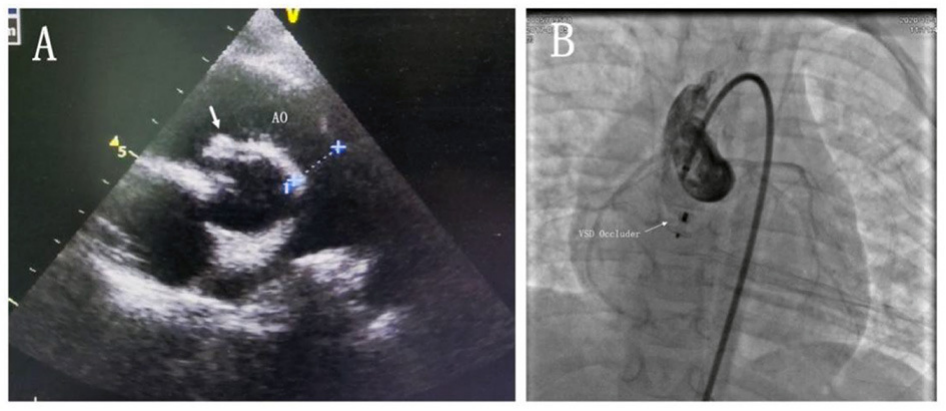
**(A)** Transthoracic echocardiogram obtained in the aortic long-axis view showing that the occluder was well (white arrow). **(B)** Cine static image front view showing the ventricular septal defect occluder in place. AO, aorta; VSD occluder, ventricular septal defect occluder.

A 3-year-old boy underwent surgical repair of ventricular septal defect (VSD) at the age of 3 months. A large perimembranous ventricular septal defect with a size of about 10 mm was detected in the child after birth, so we performed a ventricular defect repair when the child was 2 months old. Two months after surgery, transthoracic echocardiography (TTE) revealed the shunt between the aortic sinus and the right atrium without significant aortic regurgitation, showing that the diameter of the break was about 1.5 mm. Therefore, we decided to have the child undergo regular follow-up transthoracic echocardiography and observe the change in the size of the break, instead of immediate surgical intervention. TTE showed that the diameter of the break of the SOA rupture was 3 mm, accompanied by a mild-to-moderate aortic regurgitation during regular follow-ups in the hospital at 3 years after ventricular defect repair (Figures 1A,B). Upon examination, a persistent murmur was heard in the left parasternal area. Transthoracic echocardiography (TTE) showed that the diameter of the break was about 3 mm, the length of the diastolic regurgitation beam at the root of the non-coronary valve was 35 mm, and the width of the neck was 4.4 mm. The left ventricle was slightly enlarged, and the right side of the heart was normal. We decided to use the transcatheter closure (TCC) method to occlude the SVA. This was performed with the patient under general anesthesia. Right femoral 6F arterial access was obtained. A left ventricular angiogram was performed using a 5F pigtail catheter through the right femoral artery. Communication was established between NCS and the right atrium outflow tract of 3.0 mm (Figure 1C). The defect was crossed from NCS using a 5F Torcon NB Advantage Catheter (Cook Medical) and an angled hydrophilic guide wire. An 6F delivery sheath was advanced from the femoral artery and placed in the right atrium across the defect. A 5-mm VSD occluder (Shanghai Shape Memory Alloy Ltd., China) was deployed through the long sheath under fluoroscopic guidance. After the release of the VSD occluder, the occluder had good shape and position, and no substantial residual shunting was found in the aortic root angiography (Figure 2). A day later, the patient's parents were advised to give him 100 milligrams of aspirin each day and he was successfully discharged from the hospital. During the half-year follow-up, no residual shunt, embolism, infective endocarditis, or aortic reflux were observed.

## Discussion

A sinus of Valsalva aneurysm (SVA) is typically the result of a lack of fusion between the tunica media and the annulus fibrosus of the aortic valve (5). However, in the case of infective endocarditis, surgery, or trauma, it may not form SVA. In this paper, we report a rare case of SOV rupture in a child without SVA. Our patient did not experience aortic sinus rupture before or during VSD repair, and since the SOV rupture appeared 1 month after surgery, and there were no obvious indications of endocarditis, such as fever and cardiac flab formation, during this period. we speculate that the patient's previous VSD repair may have been the main cause of the SOV rupture (6, 7).

Surgery has been the main treatment for SOV rupture since 1956. The indications for surgical repair include the location of SOV and whether a rupture takes place. However, due to the implementation of sternotomy and cardiopulmonary bypass, the associated morbidity is high. Currently, there is a percutaneous closure technique, which has been proved to be a simple method with a low incidence of complications (8). In 1994, Cullen et al. described the first case of transcatheter closure of ruptured SVA, which occurred in a patient with recurrent SVA rupture. This patient developed SVA again after surgical repair (9). Fedson et al. reported the first case of transcatheter closure of ruptured aortic sinus aneurysm with an Amplatzer catheter occluder (ADO) in 2003 (10). Since then, percutaneous occluders have been used more and more. The most commonly used occluder is ADOII. Other less commonly used occluders include ventricular septal defect occluder, atrial septal defect occluder, and coils, although coils are rarely used. The venous antegrade closure technique is used for conventional ductal occluders, and the retrograde aortic approach via femoral artery is used for such devices as ventricular septal occluders and ADOII devices (11).

**Figure 3:**
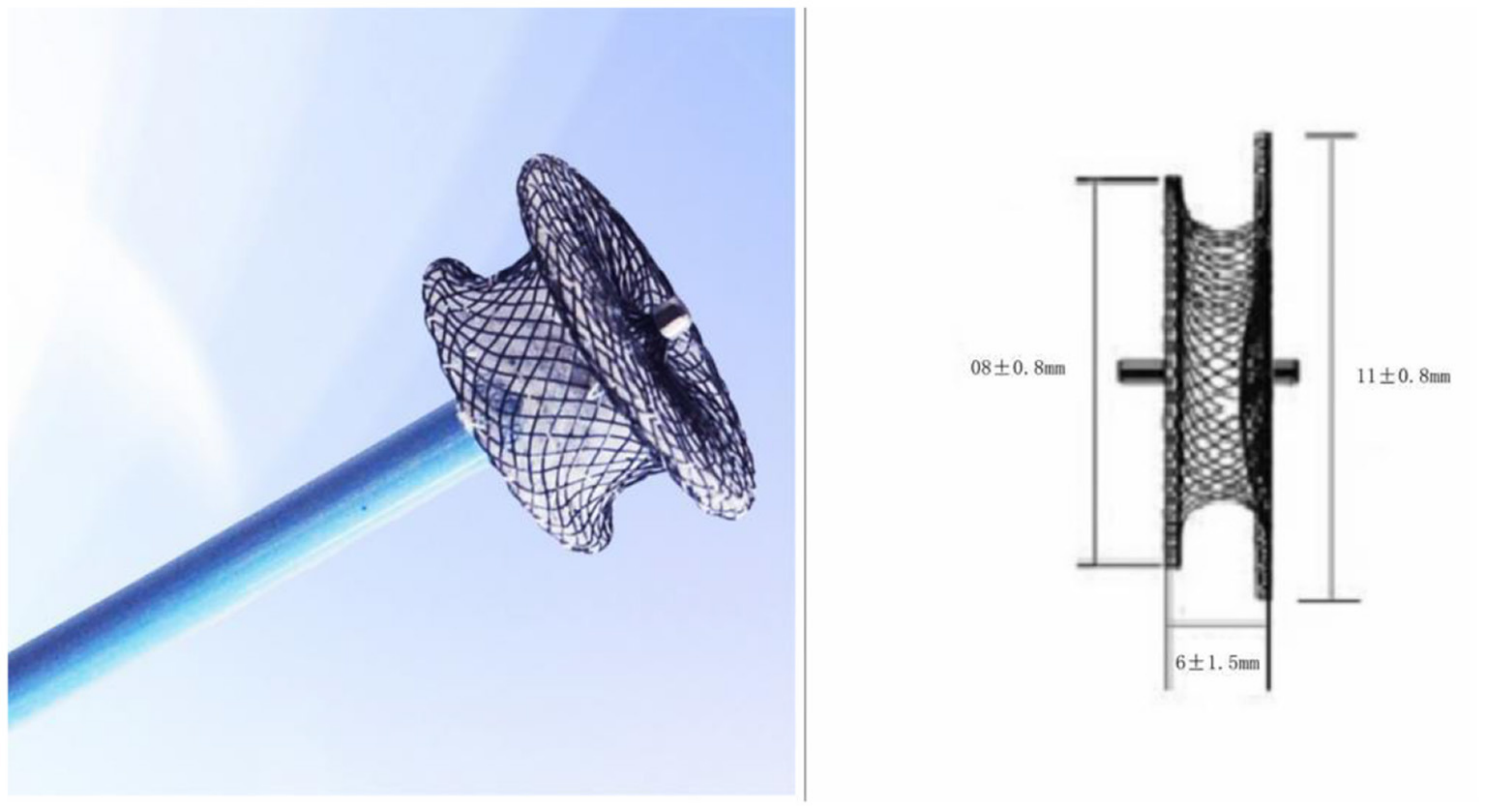
A modified double-disk ventricular occluder was used in this procedure. The left umbrella disk is slightly larger than the right umbrella disk.

In the past, few children underwent SOV surgery. The youngest one on record was 18 months old. Surgery or occlusion operation on SOV in children can prevent the formation of aortic sinus aneurysm caused by long-term pathological changes and further problems such as heart enlargement and reduction of cardiac function. Amplatzer Duct Occluder IIs (ADOIIs) are the primary tool for occlusion in children. However, ADOII is not currently available in China due to trade wars and other factors. By careful comparison, we concluded that the ventricular septal defect occluder is the most suitable alternative. The ventricular septal defect occluder is an improvement over the Amplatzer ventricular septal defect occluder, and the most important improvement is that the left umbrella disk is slightly larger than the right umbrella disk. The left umbrella disk is slightly larger than the right umbrella disk. Due to this design, the smaller right umbrella disc is located in the aortic sinus, reducing the impact on the aortic valve. This allows for the complete closure of larger defects while minimizing the impact on the aorta. This occluder can be used for any type of SOV or SVA in both adults and children (Figure 3).

In the case reported, the site of SOV rupture entered the right atrium from the non-coronary sinus of Valsalva. We used a double disc perimembranous ventricular septal defect occluder to repair SOV rupture with a retrograde method. The size of the selected device was larger than that of the aortic end of the defect: 2 mm. Because the rupture flows into the right atrium, the VSD occluder can provide stability and does not run the risk of atrial hemodynamic changes. Retrograde closure and rupture of SOV may be safer and faster than the antegrade method (12).

## Conclusion

The percutaneous closure with the ventricular septal defect occluder of ruptured SOV can be successfully performed. Percutaneous occlusion with a suitable device could be a reliable and safe alternative to surgery in small children.

## Data Availability Statement

The original contributions presented in the study are included in the article/supplementary material, further inquiries can be directed to the corresponding author/s.

## Ethics Statement

Written informed consent was obtained from the individual(s) for the publication of any potentially identifiable images or data included in this article.

## Author Contributions

TZ and SH were responsible for the diagnosis and treatment of the patient. XG and SH prepared the manuscript. JW and LW collected clinical data. All authors have read and approved the final manuscript, have agreed to be accountable for the content of the work, and contributed to the article and approved the submitted version.

## Conflict of Interest

The authors declare that the research was conducted in the absence of any commercial or financial relationships that could be construed as a potential conflict of interest.

## Publisher's Note

All claims expressed in this article are solely those of the authors and do not necessarily represent those of their affiliated organizations, or those of the publisher, the editors and the reviewers. Any product that may be evaluated in this article, or claim that may be made by its manufacturer, is not guaranteed or endorsed by the publisher.
